# *Sigesbeckia orientalis* L. Derived Active Fraction Ameliorates Perioperative Neurocognitive Disorders Through Alleviating Hippocampal Neuroinflammation

**DOI:** 10.3389/fphar.2022.846631

**Published:** 2022-03-17

**Authors:** John Man Tak Chu, Amina Abulimiti, Brian Shing Hei Wong, Guan Ding Zhao, Shi Hang Xiong, Ming Ming Zhao, Yingyi Wang, Ying Chen, Jiaqi Wang, Yan Zhang, Raymond Chuen Chung Chang, Hua Yu, Gordon Tin Chun Wong

**Affiliations:** ^1^ Department of Anaesthesiology, LKS Faculty of Medicine, The University of Hong Kong, Hong Kong, China; ^2^ Institute of Chinese Medical Sciences, State Key Laboratory of Quality Research in Chinese Medicine, The University of Macau, Taipa, China; ^3^ Laboratory of Neurodegenerative Diseases, School of Biomedical Sciences, LKS Faculty of Medicine, The University of Hong Kong, Hong Kong, China; ^4^ The State Key Laboratory of Brain and Cognitive Sciences, The University of Hong Kong, Hong Kong, China

**Keywords:** perioperative neurocognitive disorders, neuroinflammation, microglia, surgery, *Sigesbeckia orientalis* L

## Abstract

Neuroinflammation is closely related to the pathogenesis of perioperative neurocognitive disorders (PNDs), which is characterized by the activation of microglia, inflammatory pathways and the release of inflammatory mediators. *Sigesbeckia orientalis* L. (SO) is a traditional Chinese medicine which demonstrates anti-inflammatory activities in different models. In this study, we aim to isolate the active fraction from the extract of SO with higher anti-inflammatory potential and confirm if the selected fraction exerts neuroprotection against the development of PND in an animal model. Moreover, the components in the selected fraction would be determined by UPLC-PDA analysis. Three fractions were prepared by column chromatography packed with three different macroporous resins. Anti-inflammatory activities of prepared fractions were accessed in microglial BV2 cultures by nitric oxide release, gene expression of inflammatory cytokines and activation of inflammatory JNK and NF-kB pathway molecules. Our results demonstrated that the fraction prepared from D101 macroporous resin (D101 fraction) exhibited a more potent anti-neuroinflammatory effect. The neuroprotective effect of D101 fraction was further examined in postoperative mice. Our results showed that surgery-induced cognitive dysfunction was attenuated by the D101 fraction treatment. This fraction also reduced microglial activation, inflammatory cytokines and inhibiting JNK and NF-kB pathway molecules in the hippocampus. In addition, surgery induced dendritic spine loss while D101 fraction ameliorated the spine loss in the hippocampus. For safety concerns, anti-thrombotic effect was examined by tail bleeding assay and no significant change of the bleeding pattern was found. UPLC-PDA analysis indicated that flavonoids (rutin, isochlorogenic acid A, isochlorogenic acid C) and terpenoid (darutoside) were the most important components in the D101 fraction. Our results support a therapeutic, as well as the translational potential for D101 fraction in ameliorating postoperative neuroinflammation and subsequent PND in the clinical setting without increasing bleeding tendencies.

## Introduction

Neuroinflammation has been implicated in the pathogenesis of perioperative neurocognitive disorders (PNDs) such as postoperative delirium or delayed neurocognitive recovery (Subramaniyan and Terrando, 2019). Following surgery, microglial cells are activated in the central nervous system (CNS) (Hovens et al., 2015) and activation of inflammatory pathways including JNK and NF-kB pathways are observed (Ip and Davis, 1998; Liu et al., 2017). These in turn increase various proinflammatory cytokines that mediate neuroinflammatory responses and cognitive dysfunction through enhancing the transcriptional activities (Shabab et al., 2017). It is of note that a transient inflammatory response maybe beneficial in certain situations. However, it may result in significant CNS damage when the neuroinflammatory response is prolonged and this is commonly observed in various CNS disease models including PND (Subramaniyan and Terrando, 2019). Therefore, targeting microglia and neuroinflammation may have therapeutic potential in attenuating PND (Côté et al., 2012).

Chinese medicinal herbs belong to part of traditional Chinese medicine practice which are being used as a form of complementary and alternative medicine. One of the major beneficial effect is attributed to their anti-inflammatory properties (Chen et al., 2011). Siegesbeckia herba has been demonstrated to possess significant anti-inflammatory properties (Linghu et al., 2020a). *Sigesbeckia orientalis L.* (SO) is the subspecies of Sigesbeckia herba and its dried aerial part is used for managing inflammatory diseases. It has been shown that SO extract exert anti-inflammatory and analgesic effects in experimental model of arthritis (Nguyen et al., 2017; Linghu et al., 2020b), as well as in postoperative animals (Chu et al., 2018). However, the composition, as well as the molecular pathway involved in the anti-inflammatory effect of SO is largely unknown.

In present study, we have purified an active fraction from SO extract by using chromatograph column packed with macroporous resins. Anti-inflammatory capability was first examined in *in vitro* microglial BV2 culture with lipopolysaccharides (LPS). Our results revealed that fraction prepared from D101 resin column (D101 fraction) has a more potent anti-inflammatory capability. Our results also validated that D101 fraction improves cognitive dysfunction, inhibiting neuroinflammatory response and preserving synaptic structure in postoperative mice without significant impact on blood coagulating. Finally, we identified and standardized several important components in the D101 fraction. Our findings demonstrate the therapeutic, as well as the translational value of D101 fraction in PND or other neuroinflammatory diseases.

## Materials and Methods

### Chemicals and Reagents

Rutin, isochlorogenic acid A, isochlorogenic acid C and darutoside were purchased from Chengdu Pufei De Biotech Co., Ltd (Chengdu, China). Macroporous resins (AB8, D101, and S8) were purchased from Hangzhou Xingru Instrument Co., Ltd. (Hangzhou, China). Acetonitrile (ACN, HPLC grade) and methanol (HPLC grade) were purchased from RCI Labscan Limited (Thailand). Milli-Q water was prepared using a Milli-Q system. BV2 microglial cells were from Accegen (NJ, United States). DMEM/F12K medium, fetal bovine serum, penicillin/streptomycin, Alexa 488 secondary antibody and prolong gold mounting medium were purchased from Thermofisher Scientific (MA, United States). Iba1 antibody was purchased from Fujifilm Wako Chemicals (Osaka, Japan). LPS, 3-(4,5-dimethylthiazol-2-yl)-2,5-diphenyltetrazolium bromide (MTT), Phosphoric acid (analytical grade), Griess reagent, Drabkkin’s reagent, ibuprofen, paraformaldehyde and β-actin antibody were purchased from Sigma-Aldrich (MO, United States). RNAsol plus, PrimeScript RT Reagent Kit and TB Green Premix Ex Taq were purchased from Takara (Shiga, Japan). cOmplete™ protease inhibitor cocktail and PhosSTOP™ phosphatase inhibitor were purchased from Roche (Basel, Switzerland). Hito Golgi-Cox OptimStain Kit was purchased from Hitobiotec (TN, United States). IL-1β antibody was purchased from Peprotech (NJ, United States). RIPA buffer and other antibodies were purchased from Cellsignaling (MA, United States).

### Preparation of SO Extract and Fractions

*Sigesbeckia orientalis* L. (SO) was collected from Ganzhou (Jiangxi Province, China) and authenticated by Dr. Hua Yu (one of the corresponding authors). The voucher specimen (No. SO-19) was deposited at the Institute of Chinese Medical Sciences, University of Macau, Macao SAR, China.

The powdered SO (25 g) was reflux-extracted twice with 50% ethanol (1:10, w/v). The extracts were collected, filtered, and then concentrated under reduced pressure to remove the ethanol. The concentrated extract was loaded onto a macroporous resin (AB8, D101 and S8, Hangzhou Xingru Instrument Co., Ltd.; Hangzhou, China) column and allowed for statically adsorbing for 4 h. Subsequently, the column was washed with 1 L distilled water and then eluted with 1 L 25% ethanol. The collected 25% ethanol elutes were concentrated under reduced pressure and then and then lyophilized with a Virtis Freeze Dryer (The VirTis Company, New York, United States) to obtain the powdered fractions samples, namely AB8, D101 and S8 fractions. The extraction yields (%, w/w) were calculated to be 15.86, 0.98, 1.39, and 1.37% for the total extract, AB8 fraction, D101 fraction and S8 fraction, respectively.

### BV2 Microglia Cultures

BV2 microglia cells were cultured for the *in vitro* part of the study. Cells were grown in DMEM/F12K culture medium supplemented with 10% fetal bovine serum and 1% Penicillin/Streptomycin. Cells were seeded on 96 and 24 well plates respectively for subsequent assays. Cell cultures were incubated in humidified incubator at 37°C with 5% CO_2_.

### MTT Assay

Cytotoxicity of different SO fractions was first examined by MTT assay in BV2 cultures as described (Chu et al., 2016). Briefly, cells were seeded on the 96 well plates at a density of 5 × 10^4^/well. The medium was discarded after 24 h. Different concentrations of fractions were incubated with BV2 cells for 24 h. 0.5 mg/ml MTT solution was added to each well and incubated at 37°C for 4 h. Formazan salt in living cells were dissolved by 2-propanol and absorbance was measured at 570 nm by a microplate reader.

### Nitric Oxide Release Assay

BV2 cells were seeded on 24 well plates at a density of 2 × 10^5^/well. Cells were co-treated with LPS (100 ng/ml) and different SO fractions at concentrations of 0, 7.81, 15.62, 31.25, 62.5, or 125 μg/ml for 24 h. Concentrations based on the results of MTT cytotoxicity assay where the maximum dose at which over 80% of cells remain viable were chosen. Cell culture medium was collected and nitric oxide (NO) was detected by incubating 100 μl medium with 100 μl of Griess reagent for 15 min as described in the instructions. Absorbance was measured at 450 nm by microplate reader. IC50 of different fractions on NO release was calculated.

### mRNA Extraction and Real-Time PCR

Endogenous mRNA expression of different inflammatory cytokines were examined by real-time PCR as described (Chu et al., 2018). mRNAs were extracted by RNAiso plus and converted into complementary DNA (cDNA) by PrimeScript RT Reagent Kit. Expression of different inflammatory cytokines was accessed by real-time PCR with TB Green Premix Ex Taq and respective primers: 1) IL-1β, forward: CCT​CCT​TGC​CTC​TGA​TGG, reverse: AGT​GCT​GCC​TAA​TGT​CCC; 2) IL-6, forward: TTC​ACA​AGT​CCG​GAG​AGG​AG, reverse: TCC​ACG​ATT​TCC​CAG​AGA​AC; 3) TNF-α, forward: CCC​CAG​TCT​GTA​TCC​TTC​T, reverse: ACT​GTC​CCA​GCA​TCT​TGT; 4) MCP-1, forward: TGC​TGT​CTC​AGC​CAG​ATG​CAG​TTA, reverse: TAC​AGC​TTC​TTT​GGG​ACA​CCT​GCT5) GAPDH, forward: ATT​CAA​CGG​CAC​AGT​CAA, reverse: CTC​GCT​CCT​GGA​AGA​TGG.

### *In Vivo* SO Fraction Treatment and Laparotomy Procedure

**FIGURE 1:**
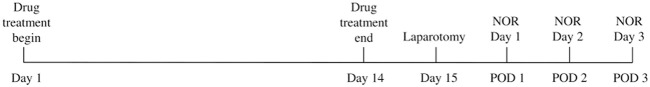
Schematic timeline of the animal experimental procedures.

3-month-old C57BL6/N mice were obtained from The University of Hong Kong. The handling of animals and all experimental procedures were conducted in accordance with National Institutes of Health guide for the care and use of Laboratory animals and Animals (Control of Experiments) Ordinance, Hong Kong, China. The use of animals was approved by the Department of Health, Hong Kong and Committee on the Use of Live Animals in Teaching and Research, The University of Hong Kong. Animals were randomly divided into 6 groups: Sham control (Ctrl), laparotomy (Lap), laparotomy with lose dose drug (LD plus Lap), laparotomy with high dose drug (HD plus Lap), drug control with high dose (HD drug) and laparotomy with ibuprofen (Ibu plus Lap). Mice were assigned to 200 μl of either D101 fraction, ibuprofen or sham PBS solution for 14 days prior to surgery. Fraction doses of 0.13 g/kg/day (high dose, HD) and 0.065 g/kg/day (low dose, LD) were based on the calculation from extraction yield and our previous report (Chu et al., 2018). Ibuprofen (60 mg/kg/day) was used as the reference control for non-steroid anti-inflammatory drug treatment. Sham control and surgery groups were given equivalent amount of PBS solution. On day 15 the mice underwent a midline laparotomy under 3% sevoflurane general anesthesia in 100% oxygen (1 L/min). After incision, the gastrointestinal tract was rubbed by fingers for 2 min and replaced back to the peritoneal cavity followed by wound closure. The mice were allowed to recover before behavioral testing. The schematic diagram of the timeline for animal experiment is illustrated in Figure 1.

### Open Field and Novel Object Recognition Test

Cognitive function was accessed by the novel object recognition (NOR) test as described (Chu et al., 2018). Briefly, on postoperative day (POD) 1, open field test was carried out. Mice were placed in an empty open field arena (40 × 40 cm) for 10 min and locomotor activities were recorded by a video recorder. Locomotor function and anxiety were accessed by the total travel distance and center duration time respectively as described (Huang et al., 2018). On POD 2, mice were placed in the same arena with two identical objects for 10 min for familiarization. On POD 3, one of the two objects was replaced with a novel object. Mice were allowed to explore in the arena for 10 min and their performance was captured by a video recorder. Object exploration time was counted when mice nose pointed towards and located within 2 cm of the object. The discrimination index was calculated by the formula: T_n_-T_f_/T_t,_ where T_n_ and T_f_ were the time of exploring novel and familiar objects, while T_t_ was the total time of exploring both objects. For the assessment of the behavioral tests, the investigator was blinded from the grouping of animals when they performed analysis on the video recording. Exclusion criteria for NOR included excessive inactivity subject of the animal as indicated by a total object interaction time < 20 s, or recognition bias on a specific side of subject during familiarization phase.

### Immunofluorescence Staining

Microglia activation in the hippocampus was examined by immunofluorescence staining. Brain tissues were fixed with 4% paraformaldehyde solution for 24 h and 20 μm frozen sections containing the hippocampal region were prepared. Non-specific binding was first blocked by 1% BSA and 0.1% of Triton X reagent in PBS. Sections were then stained with microglial marker Iba1 antibody for overnight and thereafter incubated with secondary antibody conjugated with Alexa Fluor 488 for 2 h. After incubation, sections were mounted with prolong gold medium containing DAPI. Fluorescent images in CA1 and DG regions of hippocampus were obtained using a Carl Zeiss LSM900 laser scanning confocal microscope. Analysis was performed by quantifying immunofluorescence intensity from at least four images per region.

### Multiplex Assay

Hippocampal tissues were dissected out and proteins were extracted in RIPA buffer supplemented with protease inhibitor. Inflammatory cytokine expression in the hippocampus was examined by MILLIPLEX MAP mouse cytokine/chemokine magnetic bead panels (EMD Millipore Corp. United States). All primary data points were collected on a Bio-Plex 200 system. Protein samples and detection substrates were incubated on the plate with specific antibodies-conjugated magnetic beads coated on each well. Fluorescence signal detection and analysis were performed according to the manufacturer’s protocol. Concentrations of inflammatory IL-1β, IL-6, TNF-α, MCP-1 and anti-inflammatory cytokine IL-10 in hippocampus were examined.

### Tail Bleeding Assay

Mice were fed with either 200 μl of PBS or D101 fraction (0.13 g/kg/day) for 14 consecutive days. On day 15 post feeding, tail bleeding assay was examined by Drabkin’s reagent as described (Saito et al., 2016). Briefly, mice were anesthetized with ketamine and xylazine. Distal 5 mm segment of the tail was amputated. The tail was immersed in 2.5 ml of Drabkin’s reagent. Over a total of 30 min, Drabkin’s solution was gently mixed and transferred to 96 well plate. Absorbance was measured at 420 nm by using a plate reader.

### Dendritic Spines Counting

Brains tissues were immediately removed without perfusion and rinsed with double distilled water for 2–3 s. Golgi stain impregnation and staining procedures were conducted according to the manufacturer’s protocol. After staining, coronal sections with 150 μm thickness were sectioned from the hippocampus using a cryostat. Dendritic spines in hippocampus were visualized and examined under a light microscope. The number of spines was recorded, together with the length of the dendritic segment. The results of dendritic spine density were expressed as number of spines per μm of dendrite.

### Western Blot Analysis

Protein expression in cultures and hippocampal tissues were examined by Western blot as described (Chu et al., 2018). The BV2 cells were washed with PBS and lysed with RIPA buffer supplemented with protease and phosphatase inhibitors. The hippocampal tissues were homogenized in RIPA buffer supplemented with inhibitors. Protein samples were extracted after 14,000 g centrifugation. They were resolved by SDS-PAGE and transferred onto PVDF membrane. It was then incubated with different primary antibodies at 4°C for overnight, followed by HRP conjugated secondary goat anti rabbit/mouse antibodies for 1 h. Protein band signals were visualized with enhanced chemiluminescence reagents and captured by Chemidoc imaging system (Bio-Rad, United States). Quantification of the signals was performed by densitometric analysis of the ImageJ software. Protein signals were normalized by their endogenous protein and β-actin respectively.

### Phytochemical Analysis of Active SO Fraction

Quantification of four main compounds (rutin, isochlorogenic acid A, isochlorogenic acid C and darutoside) in the active D101 fraction was performed using a Waters ACQUITY-UPLC CLASS system (Waters Corp. United States) coupled with an ACQUITY UPLC HSS T3 column (150 mm × 2.1 mm, 1.8 µm) maintained at 40°C. Samples were eluted with a mobile phase of A (0.2% H_3_PO_4_ in water) and B (0.2% H_3_PO_4_ in ACN) under a gradient program: 0–2 min, 14% B; 2–8 min, 14%–18% B; 8–10 min, 18–20%; 10–14 min, 20–25%; 14–20 min, 25–35%. The flow rate was 0.4 ml/min and the injection volume was 2 µL. Analytes were monitored at the UV wavelength of 220 nm. Between two injections, the column was washed with 100% B for 2 min and equilibrated with the initial mobile phase for 5 min.

### Statistical Analysis

Comparison between different groups of treatment was analyzed by unpaired *t* test or one-way ANOVA with Tukey *post-hoc* test. Significant differences were considered between groups when *p* < 0.05.

## Results

### Evaluation of Cytotoxicity and NO Inhibition of SO Fractions in BV2 Culture

**FIGURE 2:**
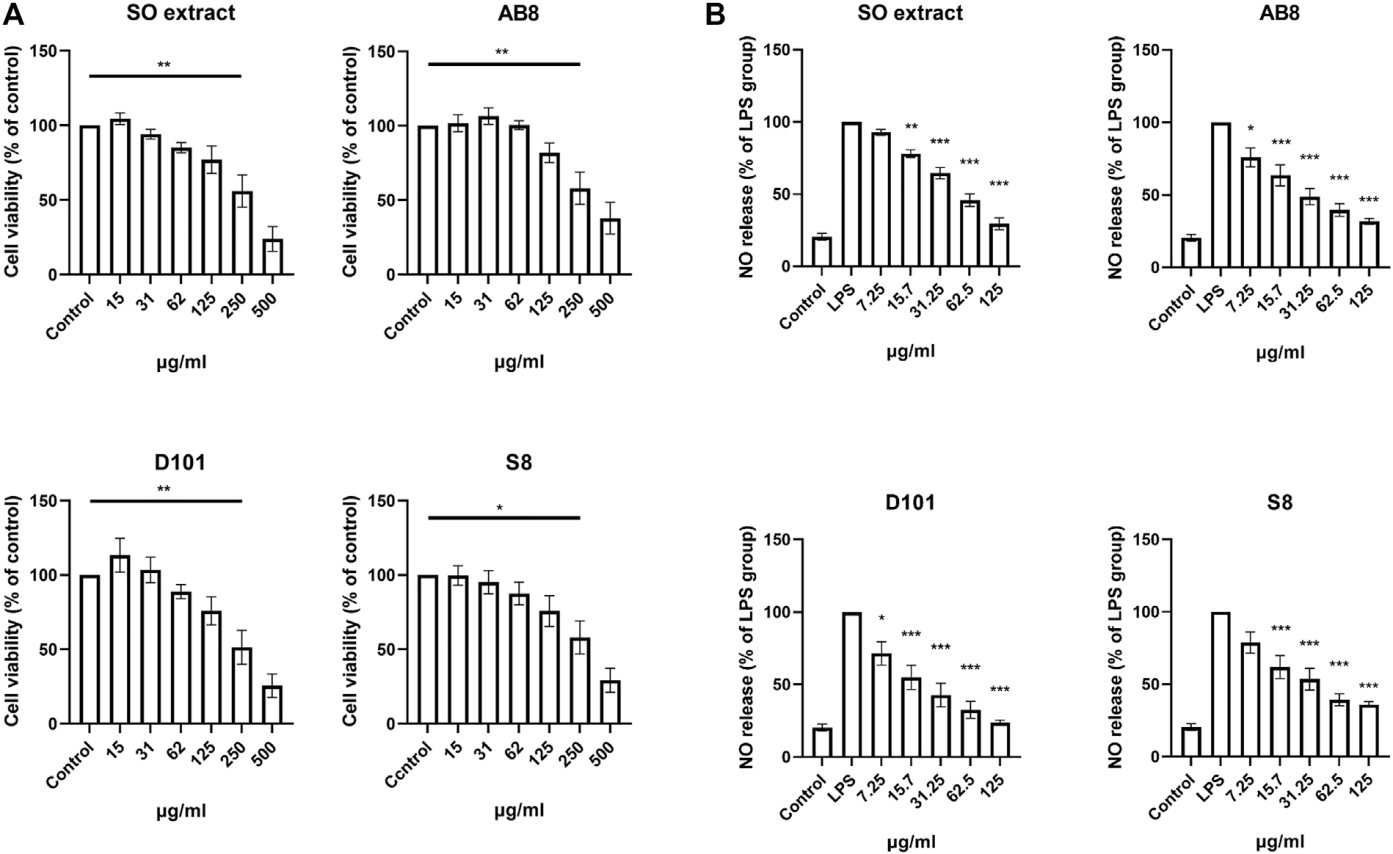
Cell viability and anti-inflammatory effects of SO fractions in BV2 cultures. **(A)** BV2 cells were treated with SO extract and fractions at different concentrations for 24 h. Cell viability was measured by MTT assay. Significant toxic effect was considered when viability was below 75% compared to control. Data are presented as the mean and SEM (*n* = 6). *p < 0.05, **p < 0.01 compared with the control group. Values were analyzed by one-way ANOVA with Tukey *post hoc* test. **(B)** Inhibition in a dose dependent manner of nitric oxide release was observed in LPS treated BV2 cultures treated with SO fractions for 24 h. Lowest IC_50_ was observed in D101 treatment group (IC_50_ = 37.32 μg/ml). Data are presented as the mean and SEM (*n* = 4). *p < 0.05, **p < 0.01, ***p < 0.001 compared with the LPS group. Values were analyzed by one-way ANOVA with Tukey post hoc test.

Three fractions from SO extract were isolated using column chromatographic approach with different macroporous resins (AB8, D101 and S8). To examine the cytotoxicity of SO extract and fractions, cells were treated with the extract and fractions for 24 h and MTT assay was performed as described (Chu et al., 2016). Significant cytotoxicity was observed at concentrations of 250 µ g/ml in both the extract and the fractions. In comparison, greater than 75% of cell viability was observed at concentrations of 125 μg/ml (Figure 2A).

To determine the NO inhibition capability of the SO fractions, BV2 cells were co-treated with LPS (100 ng/ml) and different SO fractions for 24 h. NO content was accessed by incubating the medium with Griess reagent as described (Sang et al., 2018). Significant inhibition of NO was found in cell treated with the extract and SO fractions (Figure 2B), while fraction from the D101 column showed the highest inhibitory efficacy as reflected by the lowest IC50 (Average IC50 of SO extract = 70.225 µ g/ml, AB8 = 52.885 µ g/ml, D101 = 37.32 µ g/ml, S8 = 57.58 µ g/ml). These results demonstrated a potent anti-inflammatory effect of SO fraction in LPS treated BV2 cultures, while D101 fraction may have a more potent anti-inflammatory action.

### D101 Fraction Reduced mRNA Expression of Inflammatory Cytokines and Inhibited Activation of Inflammatory NF-kB Pathways in LPS Treated BV2 Microglia

**FIGURE 3:**
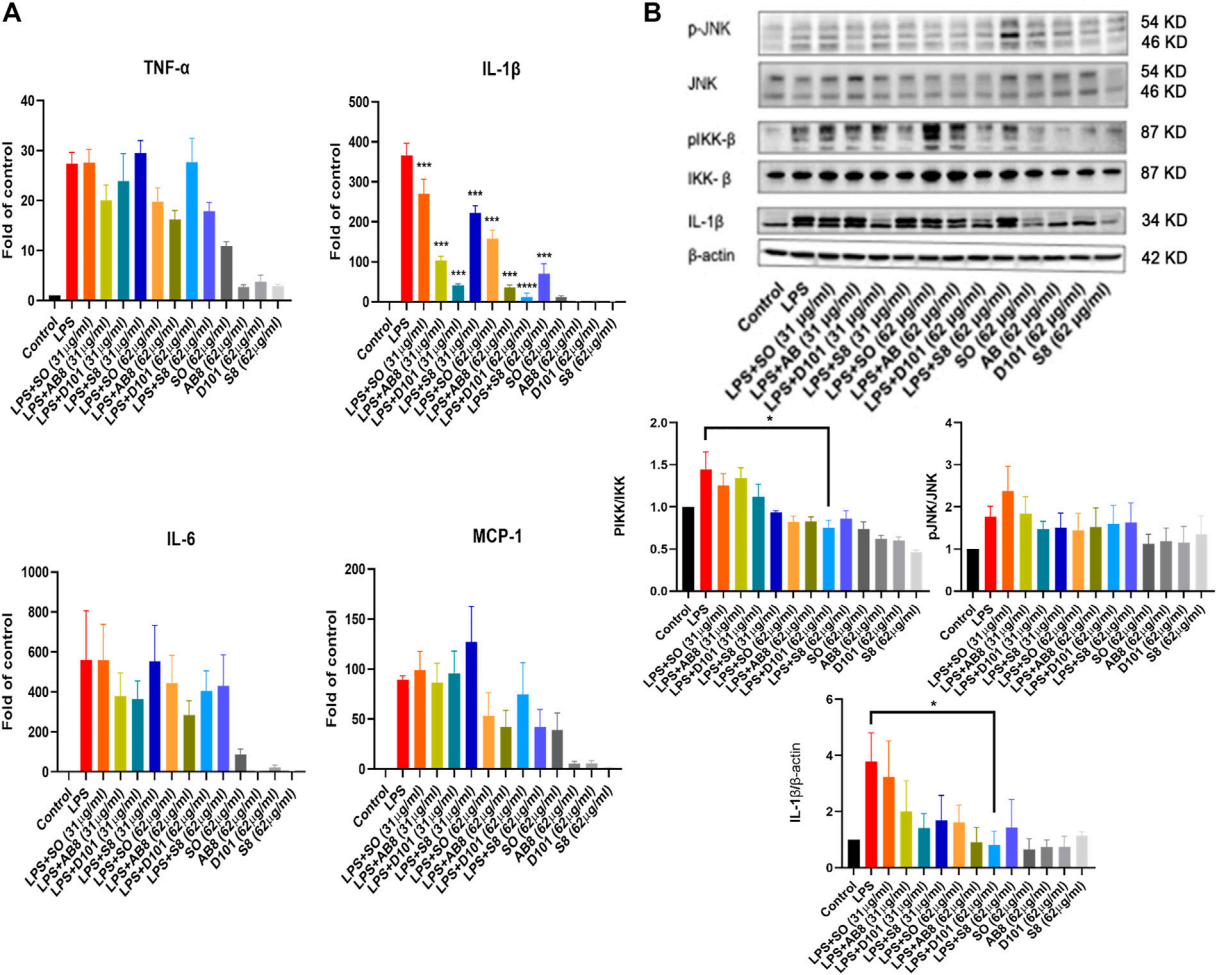
SO fractions reduced mRNA expression of inflammatory cytokines and decreased IKK phosphorylation in LPS treated BV2 cultures. **(A)** mRNA expression of proinflammatory cytokines were measured by real-time PCR. BV2 cells were co-treated with LPS and SO fractions (31 μg/ml and 62 μg/ml) for 24 h. Significant inhibition of IL-1β was observed in BV2 cultures treated with SO fractions. D101 fractions demonstrated a higher efficacy. GAPDH was used as the mRNA internal control. Data are presented as the mean and SEM (*n* = 4). ***p < 0.001 compared with the LPS group. Values were analyzed by one-way ANOVA with Tukey *post hoc* test. **(B)** BV2 cultures were co-treated with LPS and SO fractions (31 μg/ml and 62 μg/ml) for 24 h and protein expression was evaluated by Western blotting. A significant reduction of phosphorylated IKK and IL-1β was observed in D101 group (62 μg/ml). Data are presented as the mean and SEM (*n* = 3) and represents the band densities that were normalized with endogenous JNK, IKK and β-actin, respectively. *p < 0.05, **p < 0.01, ***p < 0.001 compared with the laparotomy group. Values were analyzed by one-way ANOVA with Tukey *post hoc* test.

Microglia contributes to the neuroinflammatory response by synthesizing and releasing proinflammatory cytokines. To explore the anti-inflammatory potential of different SO fractions, mRNA expression of proinflammatory cytokines was examined by real-time PCR. No significant increase in mRNA expression was observed in BV2 cultures treated with the extract and fractions alone (Figure 3A). After 24 h of LPS treatment, a drastic increase of proinflammatory cytokines was observed and SO fractions significantly inhibited IL-1β (Figure 3A). Among the fractions, D101 was shown to have a stronger inhibitory efficiency.

To examine if the SO fractions inhibit inflammatory NF-kB and JNK pathways, phosphorylation of IKK and JNK molecules were analyzed by Western blot. LPS significantly enhanced the expression of phosphorylated JNK and IKK, while no significant modulation of phosphorylated JNK was observed in BV2 cells (Figure 3B). In contrast, a significant reduction in phosphorylated IKK expression was found in BV2 cultures treated with D101 fraction (Figure 3B). Taken together, these data suggested that D101 fraction has a more potent anti-neuroinflammatory activities compared with other fractions and extract.

### D101 Fraction Treatment Attenuated Cognitive Dysfunction After Surgery

**FIGURE 4:**
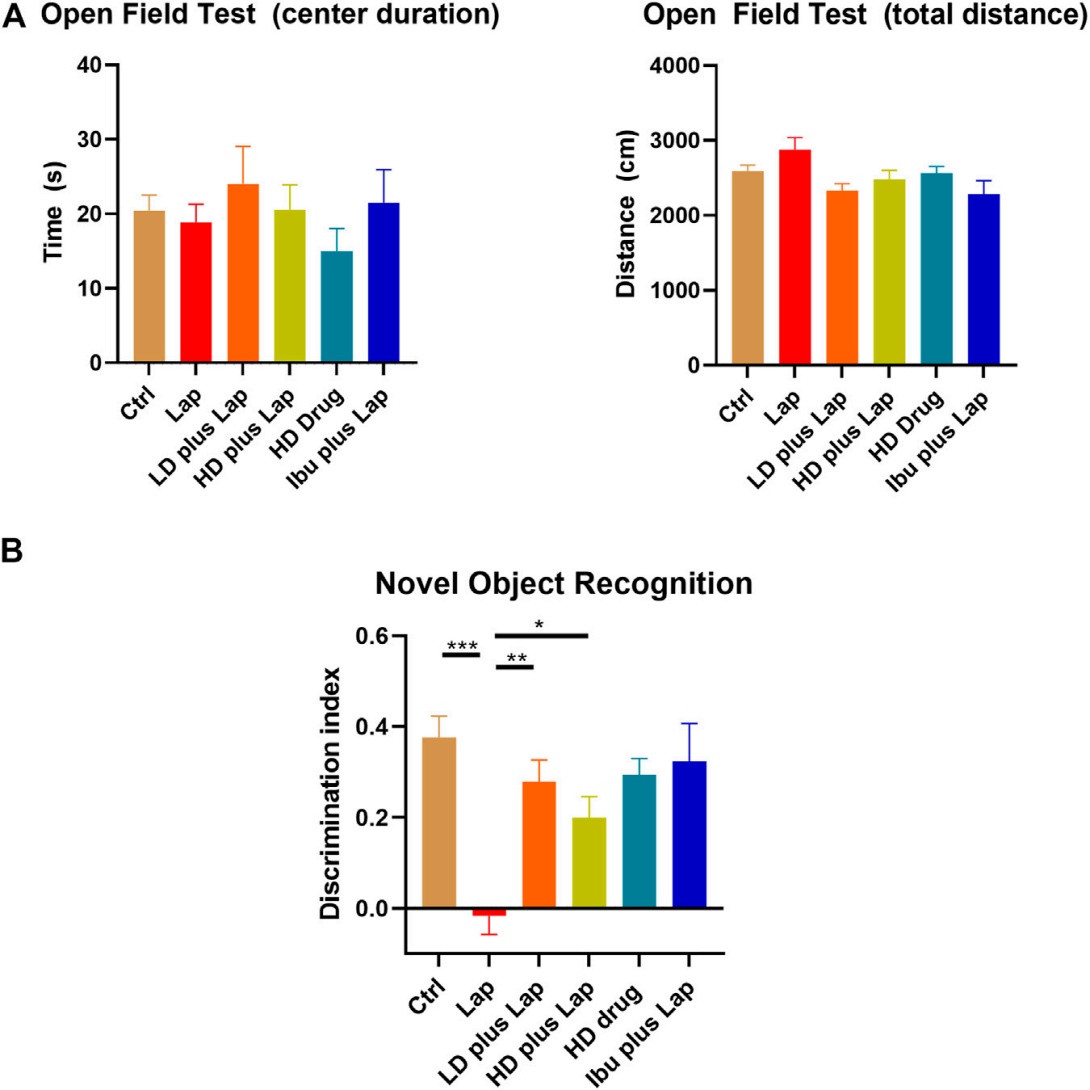
D101 treatment improved cognitive function in postoperative animals. **(A)** Open field test on POD 1. No significant anxiety was observed in all animal groups. **(B)** Novel object recognition test on POD 3. Significant increase in the discrimination index was shown in laparotomy group after D101 treatment. Data are presented as the mean and SEM (*n* = 5–6). *p < 0.05, **p < 0.01, ***p < 0.001 compared with the laparotomy group. Values were analyzed by one-way ANOVA with Tukey *post hoc* test.

In view of the anti-neuroinflammatory characteristics of the D101 fraction, we further evaluated the neuroprotective of D101 fraction in our established laparotomy model (Huang et al., 2018). Locomotor activities, anxiety and cognitive function were first examined by the open field and NOR test. In open field test, no significant modulation of locomotor function (travel distance) and anxiety (center duration time) was observed in all animal groups (Figure 4A). In NOR test, significant reduction in the discrimination index value was observed in the laparotomy group compared with control, indicating that surgery induced cognitive dysfunction (Figure 4B). The reduction was reversed by D101 fraction pretreatment in postoperative mice (Figure 4B). These data implicated that D101 improved cognitive function in postoperative animals.

### D101 Fraction Treatment Reduced Microglial Activation in the Hippocampus After Surgery

**FIGURE 5:**
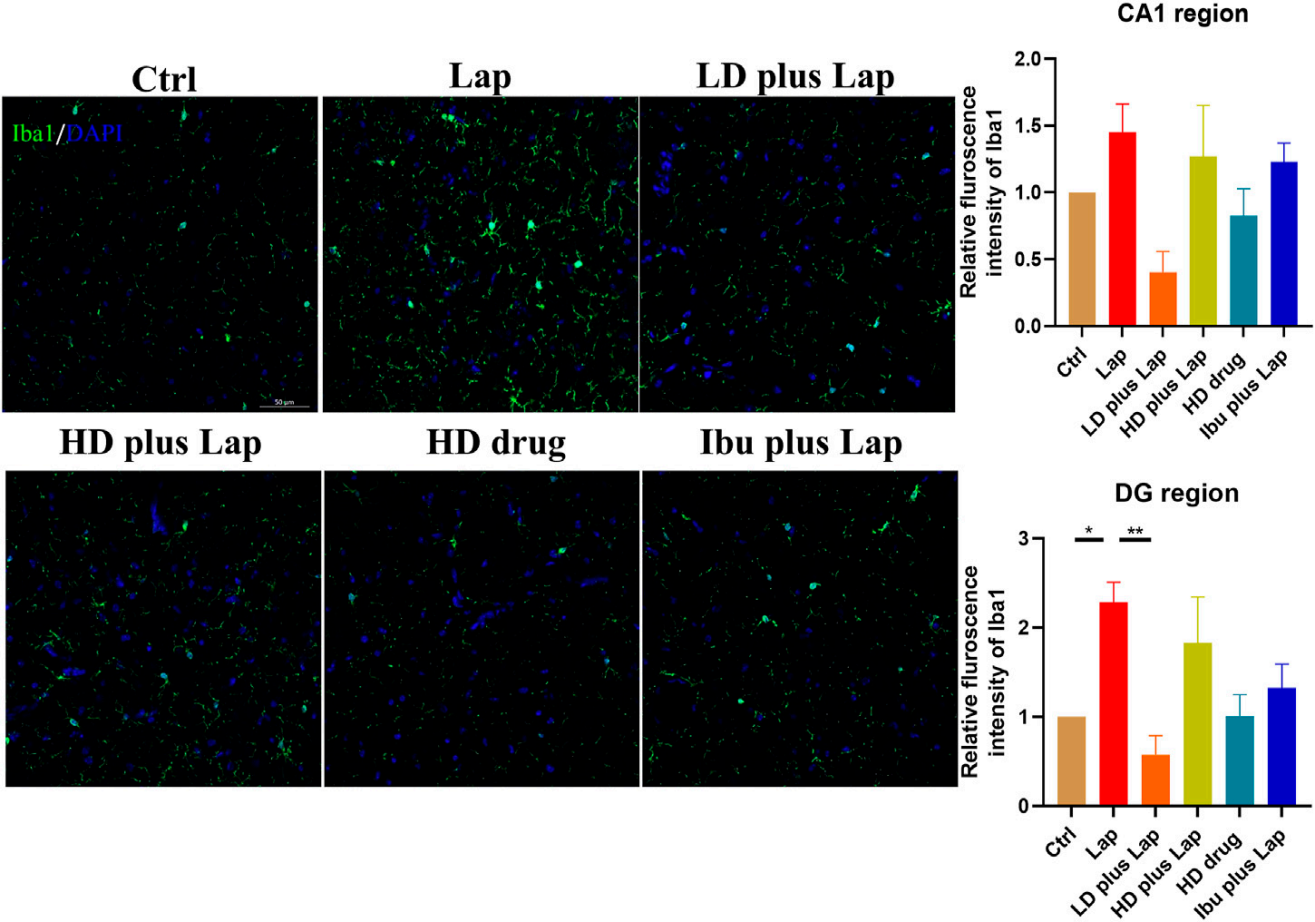
D101 fraction reduced microglia activation in the hippocampus after surgery. Left panel shows the representative images of merged Iba1 and DPAI immunofluorescence signals in the hippocampus. Immunofluorescence intensity of Iba1 in CA1 and DG regions was evaluated by confocal microscopy. Up-regulation of Iba1 immunofluorescence signals were observed in the DG region of laparotomy group while D101 treatment reduced the microglial activation after surgery. Scale bar = 50 µm. Data are presented as the mean and SEM (*n* = 4). *p < 0.05, **p < 0.01 compared with the laparotomy group. Values were analyzed by one-way ANOVA with Tukey *post hoc* test.

Microglia activation is a prominent feature in various cognitive dysfunction model and correlates to the pathological changes in the hippocampus (Dheen et al., 2007). The microglia-specific marker Iba1 was used to examine the activation of microglia in the hippocampus. No significant change of Iba1 intensity in CA1 region was observed among all groups (Figure 5). However, in DG, significance increase of Iba1 positive immunofluorescence was observed in the laparotomy group while Iba1 intensity was reduced in the laparotomy group with D101 fraction pretreatment (Figure 5). These results indicated that D101 fraction reduced microglia activation in the hippocampus of postoperative mice.

### D101 Fraction Treatment Reduced Inflammatory Cytokines and Inflammatory Pathways Activity in the Hippocampus After Surgery

**FIGURE 6:**
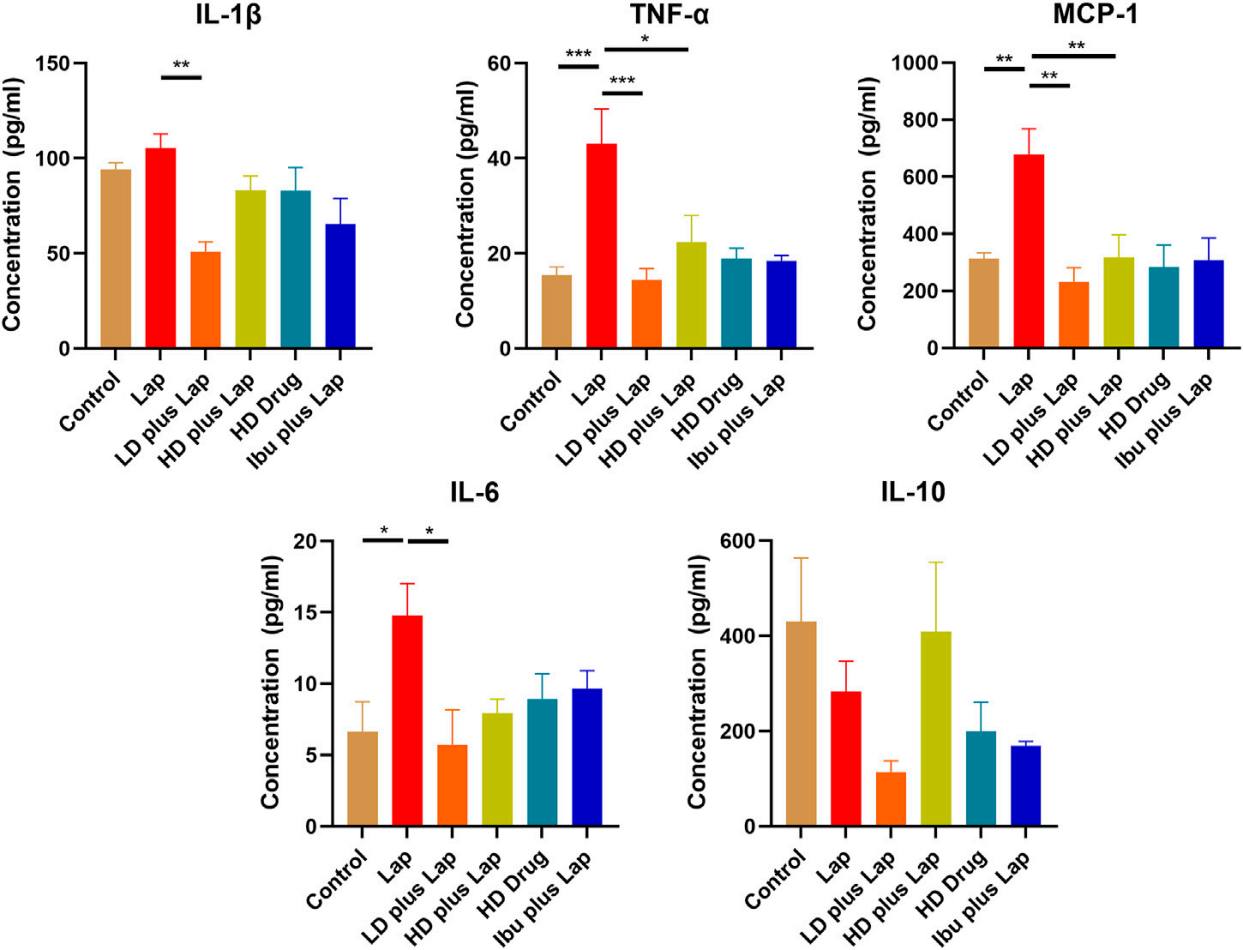
D101 fraction reduced inflammatory cytokine expressions in the hippocampus of postoperative mice. Protein expression of different inflammatory cytokines in the hippocampus were examined. A significant increase in cytokines was observed in the hippocampus after surgery while inhibition of these cytokine expression was observed in D101 fraction treated group. Data are presented as the mean and SEM (*n* = 4). *p < 0.05, **p < 0.01, ***p < 0.001 compared with the laparotomy group. Values were analyzed by one-way ANOVA with Tukey *post hoc* test.

**FIGURE 7:**
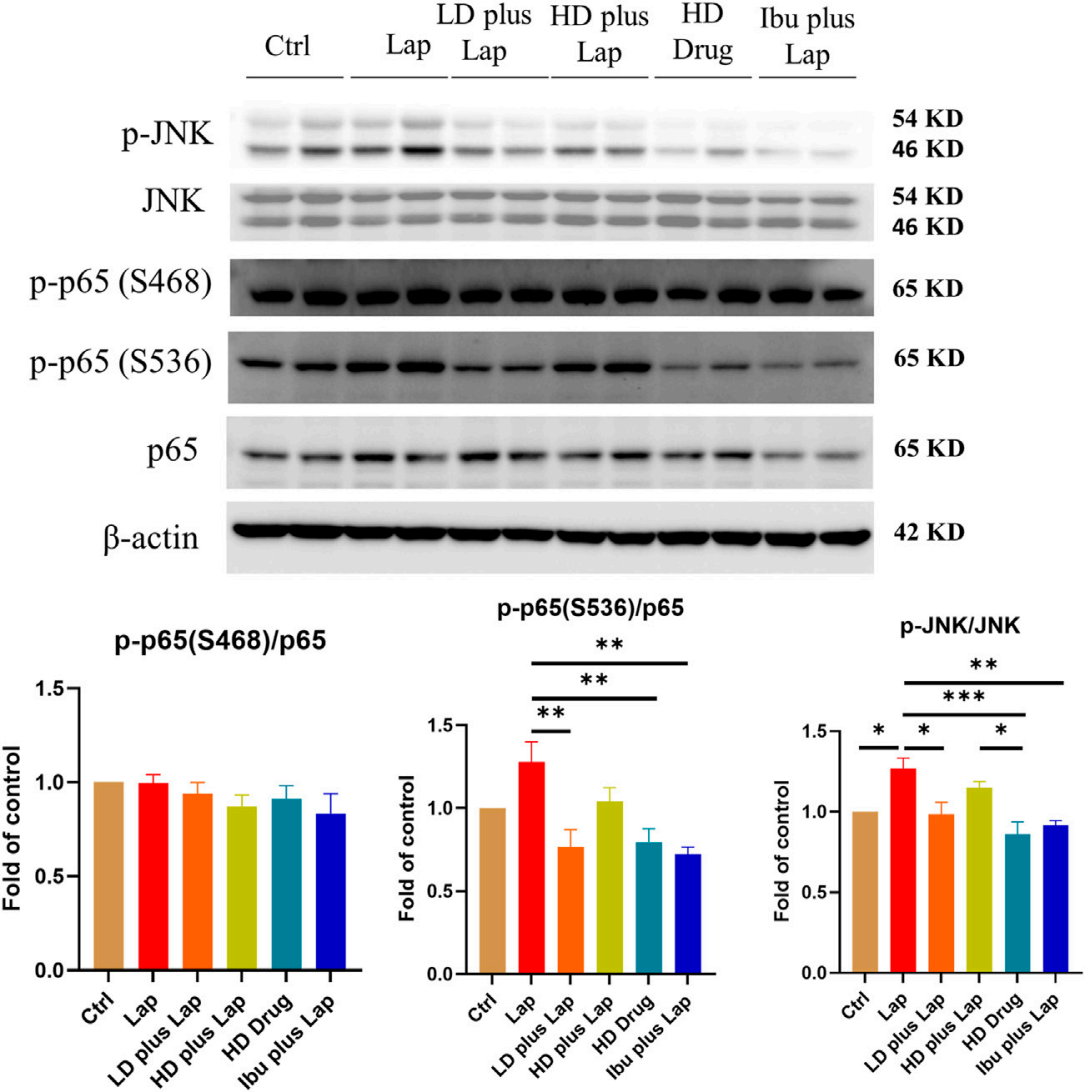
D101 fraction inhibited activation of JNK and NF-kB in the hippocampus of postoperative mice. Inhibition of JNK and NF-kB pathway after D101 fraction treatment in postoperative mice. An increase in phosphorylated JNK and p65 was found in the hippocampus after surgery and D101 fraction significantly inhibited this up-regulation. Data are presented as the mean and SEM (*n* = 4–5) and represents the band densities that were normalized with endogenous JNK, p65 and β-actin ,respectively. *p < 0.05, **p < 0.01, ***p < 0.001 compared with the laparotomy group. Values were analyzed by one-way ANOVA with Tukey *post hoc* test.

The inflammatory cytokine profiles and the activities of JNK and NF-kB pathway in the hippocampus of postoperative mice were evaluated. Results from the Multiplex assay indicated a significant up-regulation of a panel of inflammatory cytokines in laparotomy group (Figure 6). With D101 fraction treatment, the expression of inflammatory cytokines including IL-1β, TNF-α, MCP-1 and IL-6 was inhibited in the laparotomy group (Figure 6). However, no significant modulation was observed in the anti-inflammatory IL-10 (Figure 6). JNK and NF-kB pathway activities in the hippocampus were also examined by Western blot. Significant up-regulation of phosphorylated JNK and p65 was observed in the laparotomy group, implicating the activation of the two inflammatory pathways in the hippocampus (Figure 7). Consistent with the Multiplex assay results, D101 fraction inhibited phosphorylated JNK and p65 expression in postoperative mice (Figure 7). These findings, together with the *in vitro* data, supported the neuroprotective effect of D101 fraction in postoperative animals by targeting microglia related neuroinflammation.

### D101 Fraction Prevented Surgery Induced Dendritic Spines Loss in the Hippocampus

**FIGURE 8:**
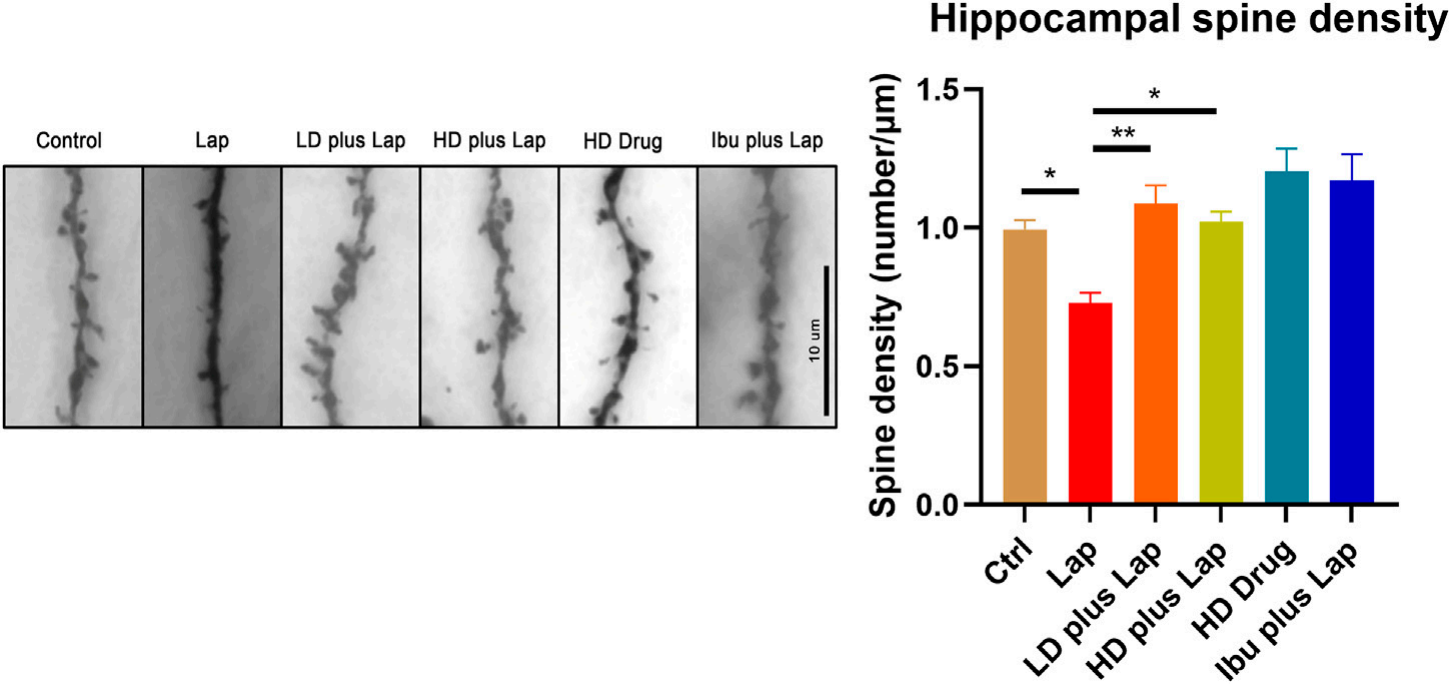
D101 fraction preserved dendritic spine density in the hippocampus of postoperative mice. Dendritic spines in the hippocampus were visualized by Golgi staining. Left panel shows the representative images of dendritic spines in hippocampus. Decrease of spine density was observed in the hippocampus after surgery. With D101 fraction treatment, significant increase of spine density was found in postoperative mice. *n* = 10 in total, 2–3 dendrites were chosen at random from four mice in each group. Data are presented as the mean and SEM. *p < 0.05, **p < 0.01 compared with laparotomy group. Values were analyzed by one-way ANOVA with Tukey post hoc test.

Dendritic spines were visualized by Golgi stain and the number of spines in the hippocampus were counted. Dendritic spine reduction in the hippocampus was observed in the laparotomy group (Figure 8). On the other hand, significant increase of the number of spines was observed in postoperative mice treated with D101 fraction. These data suggested that D101 fraction preserved synaptic structure in postoperative mice, with the possibility through inhibiting neuroinflammatory response.

### No Significant Effect on Bleeding Pattern Was Observed in D101 Fraction Treated Mice

**FIGURE 9:**
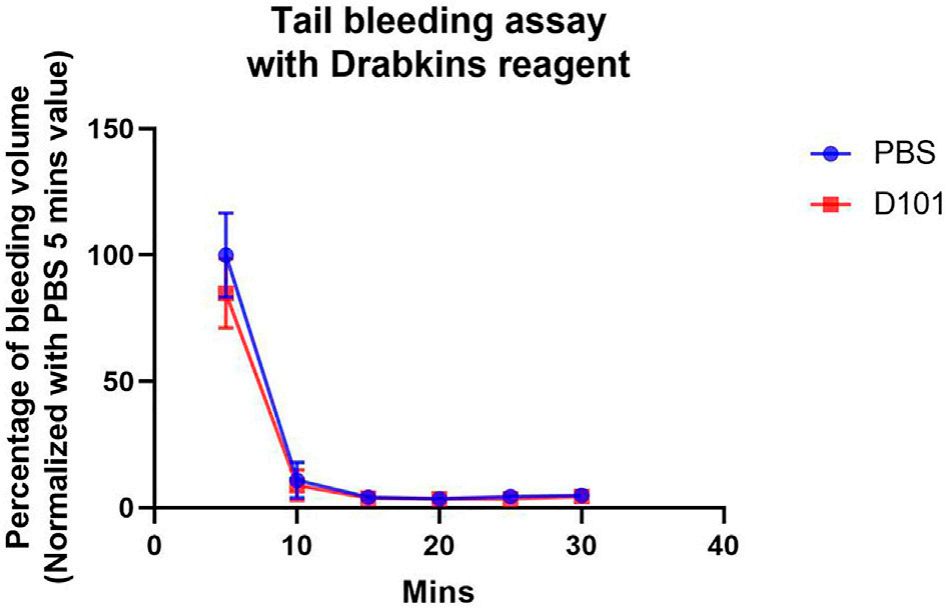
No significant effect on coagulation was observed in C57BL6/N mice treated with the D101 fraction. C57BL6/N mice were treated with either PBS or D101 fraction for 14 consecutive days and the bleeding pattern was accessed. No significant anti-coagulating pattern was observed in mice with D101 fraction treatment. Data are presented as the mean and SEM (*n* = 3). Values were analyzed by unpaired *t* test in each time interval.

To explore the potential use of the D101 fraction in the clinical setting, the effect of D101 on haemostasis were examined by tail bleeding assay. Our data showed that there was no significant increase of hemoglobin loss between vehicle and D101 treated groups (Figure 9). The bleeding pattern was similar that major blood loss was seen in the first 5 min. Coagulation and wound healing were observed in both groups at 10 min interval and minimum bleeding was observed at later time points. These data suggested that hemostatic capability was not significantly affected by exposure to the D101 fraction.

### Chemical Analysis of D101 Fraction

**FIGURE 10:**
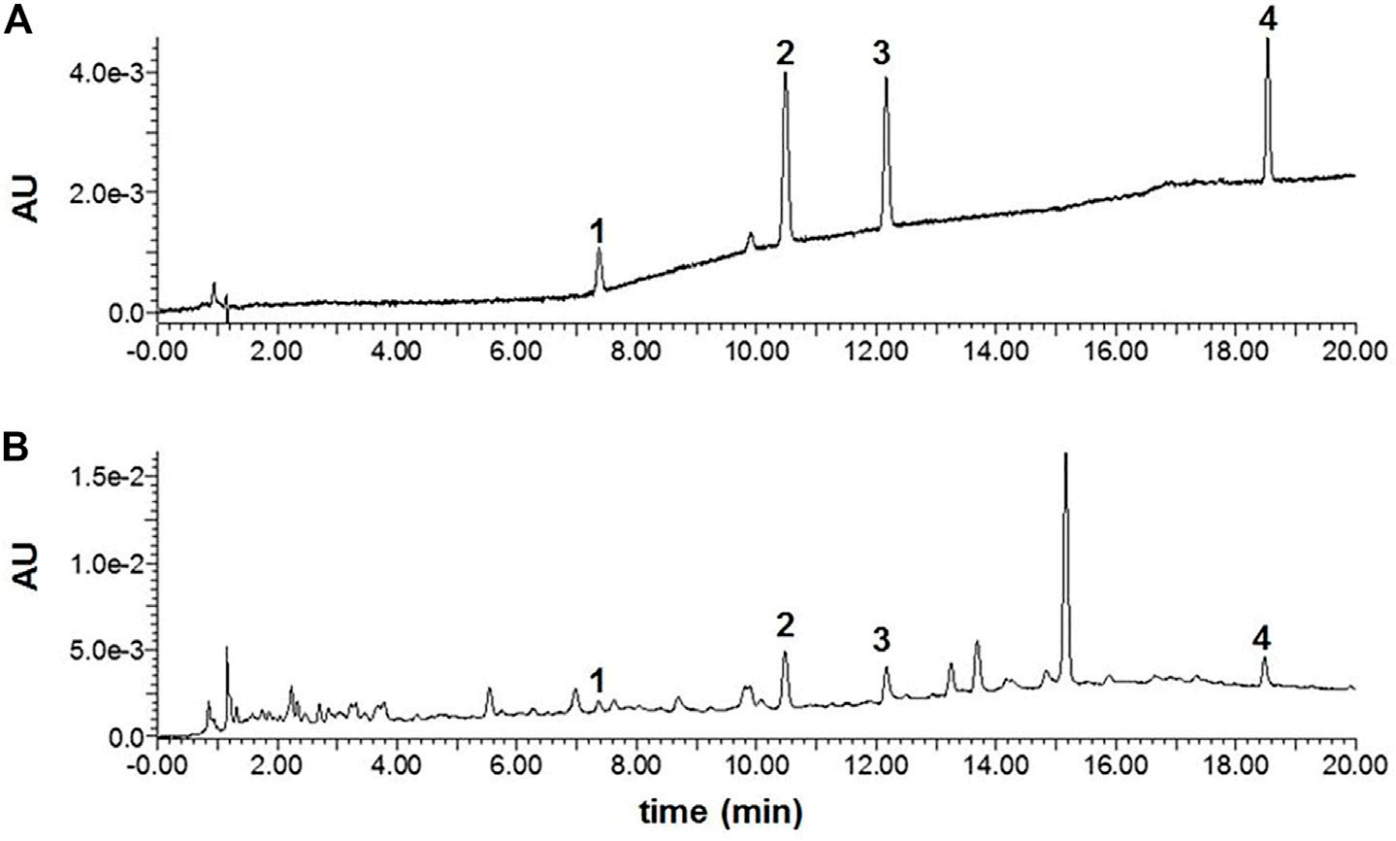
The UPLC-PDA chromatograms of mixed standards **(A)** and the D101 fraction **(B)**. 1: rutin, 2: isochlorogenic acid A, 3: isochlorogenic acid C, and 4: darutoside.

From the above results, we have shown that D101 fraction has potential to be used as part of an anti-neuroinflammatory strategy. Therefore, the chemical constitutions of the D101 fraction were analyzed using the UPLC-PDA approach. The chromatograms of the D101 fraction and the mixed standards (1: rutin, 2: isochlorogenic acid A, 3: isochlorogenic acid C, and 4: darutoside) are illustrated in Figure 10. All the quantified compounds can be well chromatographically separated from other interferences with the current developed UPLC-PDA assay method. Moreover, by using the corresponding chemical standards, the contents of three compounds including rutin, isochlorogenic acid A, isochlorogenic acid C and darutoside in the fraction were quantified to be 0.24 ± 0.02, 0.47 ± 0.05 0.59 ± 0.06, and 3.38 ± 0.30%, respectively.

## Discussion

PND is closely related to the microglia activation and neuroinflammatory response after surgery (Hovens et al., 2014). *Sigesbeckia orientalis* L. has long been used for treating inflammatory diseases and our recent report also demonstrates that the SO extract ameliorates neuroinflammatory responses in postoperative animals (Chu et al., 2018). In this report we have further purified the active fraction from the extract by using the column chromatographic approach with different macroporous resins (AB8, D101 and S8) and characterized their anti-neuroinflammatory properties. Among these fractions, we have first shown the promising results in the fraction prepared from the D101 macroporous resin column in BV2 cultures. We used BV2 cell line primarily as a screening tool as they share similarities with primary microglia. However, they may differ from primary cells in aspects of their transcriptional profile and physiological functions and have crucial difference in secretion and gene expression when challenged with inflammatory stimulus such as LPS or chemoattractant (He et al., 2018). To address this limitation, we further validated the effects of D101 fraction in our *in vivo* animal model. In line with our *in vitro* findings, the D101 fraction exerted an anti-neuroinflammatory, as well as neuroprotective effect in mice after laparotomy. Reduction in microglia activation, inflammatory cytokines and inflammatory pathway activation were also observed in postoperative mice treated with D101 fraction. Finally, no significant effect on haemostasis was seen in mice with D101 fraction treatment. These data support a translational potential of D101 fraction as an alternative against microglia related neuroinflammation and cognitive dysfunction.

After surgery, it is well recognized that a transient inflammatory response is beneficial for tissue repairing and combating infection (Koh and DiPietro, 2011). However, it can also lead to microglia activation and neuroinflammation (Huang et al., 2018). Microglia are the resident macrophages of the CNS and they play key roles in diverse physiological function such as regulating neurotransmitters, neurotrophic factors, and inflammatory mediators for supporting neuronal and synaptic activities (Kettenmann et al., 2011). They are equipped with different molecular sensors to fulfil their role in maintaining CNS homeostasis. In other cognitive disorder models such as Alzheimer’s disease (AD), resting microglia are stimulated by the amyloid deposit (Jana et al., 2008). These overactivated microglia will contribute to the neuroinflammatory environment which induces subsequent neuronal dysfunction (Perry et al., 2010). Moreover, it has been shown that astroglial cells can also be activated by surgical trauma through diverse mechanism (Feng et al., 2017; Liu and Yin, 2018). Our previous findings add more evidence by demonstrating that laparotomy triggers microglial activation in the hippocampus of 3-month-old mice (Huang et al., 2018). Strategies targeting microglial activation and related neuroinflammation remains a potential therapeutic option to combat PND and our current findings indicated that D101 fraction from SO is a suitable candidate for clinical evaluation.

We first compared and characterized the anti-neuroinflammatory properties of SO fractions in LPS treated BV2 cultures. In the CNS, NO can be released from activated microglia and over production of NO is a characteristic of microglia activation and neuroinflammatory response (Sharma et al., 2007; Yuste et al., 2015). Our *in vitro* results demonstrated a general NO inhibitory effect of all SO fractions in LPS-treated BV2 microglia (Figure 2), thus supporting the anti-neuroinflammatory effect of SO. By comparing the IC50s of NO release, we demonstrated that the D101 fraction had a higher efficacy in inhibiting NO release compared with the extract and other fractions (Figure 2). *In vivo* results from Iba1 staining indicated the inhibitory effect of D101 fraction on microglia activation, along with the improvement of hippocampal dependent recognition memory in postoperative animals (Figures 4, 5). These findings suggest that D101 fraction impedes microglia activation and improve cognitive function in postoperative animals.

It has been shown that microglia are the source of inflammatory cytokines and its activation correlates to the secretion of interleukin cytokines, tumor necrosis factor and monocyte chemoattractant protein (Hanisch, 2002). Their pathological roles are highlighted in cognitive impairment (Liu and Yin, 2018). For instance, up-regulation of IL-1β and TNF-α was observed in the postoperative animals and induce cognitive dysfunction (Cibelli et al., 2010; Terrando et al., 2010). TNF-α and IL-1β are shown to be involved in abnormal synaptic function (Heir and Stellwagen, 2020), mitochondrial function (Batista et al., 2021) and long term potentiation/depression (Hoshino et al., 2017; Heir and Stellwagen, 2020). MCP-1 is shown to be up-regulated in hippocampus after surgery and it contributes to macrophages infiltration which could amplify the neuroinflammatory condition (Feng et al., 2017; Liu and Yin, 2018). In post-mortem brains of AD patients, increase in these cytokines and their respective receptors are frequently observed and is correlated to the neuropathological changes in the hippocampal region (Kinney et al., 2018). In our BV2 culture model, SO extract and fractions were shown to be effective in inhibiting IL-1β expression (Figure 3) and D101 was shown to have higher inhibiting efficacy. Subsequent validation in animal model also showed that D101 fraction treatment inhibited cytokine protein expressions in the hippocampus after surgery (Figure 6). These findings indicate the anti-neuroinflammatory capability of D101 fraction targeting microglia related neuroinflammation in both cell and animal models. Nevertheless, no modulatory effect of either surgery or D101 fraction was observed with the anti-inflammatory IL-10 (Figure 6). IL-10 is a pleiotropic cytokine characterized by its potent immunosuppressive effect which limits the overactivation of inflammatory response (Iyer and Cheng, 2012). Surprisingly, neither surgery or D101 fraction influenced expression of this anti-inflammatory cytokine, implying that the overwhelming neuroinflammatory response may not be related to the imbalance of proinflammatory and anti-inflammatory pathway activities and D101 fraction is not capable on modulating IL-10 related anti-inflammatory pathway.

To elucidate how the D101 fraction could regulate the production of proinflammatory cytokines, we investigated if the activation of inflammatory signaling cascade was modulated. It has been demonstrated that regulation of inflammatory mediators is closely related to the inflammatory JNK and NF-kB pathways (Wang et al., 2012; Shih et al., 2015). Both JNK and NF-kB can be activated (phosphorylated) by external inflammatory stimulus such as reactive oxygen species (Morgan and Liu, 2011) and infection (Clarke et al., 2001; Hiscott et al., 2001). This phosphorylation can facilitate the translocation of their downstream transcriptional factor and enhance the transcriptional activities of inflammatory cytokines (Roy et al., 2008; Shabab et al., 2017). In postsurgical animal models, JNK and NF-kB were also shown to be activated in the hippocampus and contribute to neuroinflammation and cognitive dysfunction (Vacas et al., 2013). In contrast, inhibition of either pathway exerts neuroprotective effects in various cognitive dysfunction models (Nijboer et al., 2010; Saggu et al., 2016). Our results demonstrated that phosphorylation of both JNK and NF-kB occurred in microglia and brain after LPS and surgery (Figures 3, 7). As expected, D101 fraction was shown to be effective in reducing phosphorylation of those molecules in microglia cells and hippocampus (Figures 3, 7), thus implicating the possibility that the D101 fraction reduces the neuroinflammatory response by impeding the activation of inflammatory NF-kB or JNK pathways.

We further investigated if D101 fraction could preserve synaptic structure by examining synaptic density. Synaptic loss precedes to neuronal cell death and subsequent cognitive dysfunction (Brouillette, 2014). Previous studies have shown that neuroinflammation triggers the synaptic pruning and results in the loss of dendritic spines (Ransohoff et al., 2015). Also, dendritic spine density and complexity is correlated to the cognitive function in different neurodegeneration models whereas therapeutic treatment leading to the attenuation of cognitive dysfunction accompanies with the increase of dendritic spine density (Reza-Zaldivar et al., 2020). As cognitive improvement and inhibition of neuroinflammatory response were observed in postoperative mice with D101 fraction treatment, we proceeded to investigate if D101 fraction could ameliorate cognitive dysfunction through preserving dendritic spines. Results of Golgi staining showed that surgery reduce the dendritic spine number while D101 fraction treatment partially prevented the dendritic spine loss in the hippocampus (Figure 8). These data support our findings that D101 fraction inhibits neuroinflammatory response and minimizing subsequent dendritic spine loss, thus attenuating cognitive dysfunction in postoperative mice.

The potential detrimental side effects of herbal medicine is an obvious concern when considering their use in the perioperative setting, especially the risk of bleeding (Wong and Townley, 2010). Some of the common herbs such as Gingko and Ginseng are potent inhibitors of platelet activation and increase the risk of bleeding during surgery (Wong and Townley, 2010). To examine if the D101 fraction affect bleeding pattern, tail bleeding assay were performed. The results demonstrated that administration of D101 fraction neither modulated the bleeding pattern nor the bleeding volume compared with the sham control group, thus showing the translational value of D101 fractions into clinical use targeting surgery induced neuroinflammation and PND.

In summary, we have isolated the active fraction from the extract of *Sigesbeckia orientalis* L. with enhanced anti-inflammatory capability. And the possible chemical compound profile responsible for the anti-inflammatory effects is identified. Among different fractions, the one purified from the D101 column has a more prominent anti-inflammatory effect and the neuroprotective effects are confirmed in the hippocampus of postoperative mice. Furthermore, no significant gross effect on bleeding was observed in mice with D101 fraction treatment. These data strongly support a translational potential of using purified D101 fraction not only in perioperative neuroinflammation, but also in other neurodegenerative diseases such as Alzheimer’s disease or multiple sclerosis, which are also associated with microglial dysfunction and neuroinflammation.

## Data Availability

The raw data supporting the conclusion of this article will be made available by the authors, without undue reservation.
